# The Science and Art of Surgery

**Published:** 1878-05

**Authors:** 


*The Science and Art of Surgery.* Being a Treatise on Surgical Injuries, Diseases and Operations, by JOHN ERIC ERICHSEN, F. R.S., F.R.C.S. Revised by the author from the seventh English edition. Illustrated with 862 engravings. Two volumes. Philadelphia: Henry C. Lea. 1878. Buffalo: T. H. Butler. Price, in cloth $8.50; leather, $10.50.

This author is quite a favorite with American as well as English readers. His carefulness of statement, particularity in treatment of subjects and conservatism of judgment commend his work.

In the matter of ether and chloroform, Erichsen is not a devotee to one or the other. His judicious treatment of this subject indicates balance of mind, and his advice in the matter is as usual, intelligent and conservative.

Injuries and shock to the spine are considered more at length here than we remember to have seen in other works. The subjects of Lithotomy and Lithotrity are very well presented, and the practice which is now prevailing more and more of using Allarton's method, and of making perineal lithotrity, is given suitable attention. In the matter of amputations near the ankle and at the knee some good sections are afforded.

